# The Role of Oxidative Stress and Total Antioxidant Capacity in the Management of Impacted Third Molars: A Narrative Review

**DOI:** 10.3390/dj14010044

**Published:** 2026-01-08

**Authors:** Isis Mateos-Corral, Rogelio González-González, Marcelo Gómez Palacio-Gastelum, Ronell Bologna-Molina, Sandra López-Verdín, Omar Tremillo-Maldonado, Victor H. Toral-Rizo, Nicolás Serafín-Higuera

**Affiliations:** 1 Department of Oral Surgery, School of Dentistry, Autonomous University of Baja California, Mexicali 21040, Mexico; isis.mateos@uabc.edu.mx; 2 Research Department, School of Dentistry, Juarez University of the Durango State, Durango 34000, Mexico; rogelio.gonzalez@ujed.mx (R.G.-G.); mgpg@ujed.mx (M.G.P.-G.); ronell.bologna@ujed.mx (R.B.-M.); omar.tremillo@ujed.mx (O.T.-M.); 3 Molecular Pathology Area, Diagnostics in Pathology and Oral Medicine School of Dentistry, University of the Republic, Montevideo 11600, Uruguay; 4 Comprehensive Dental Clinics Department, Research Institute of Dentistry, University Center of Health Sciences, University of Guadalajara, Guadalajara 44340, Mexico; sandra.lverdin@academicos.udg.mx; 5 Orocenter Clinic, Faculty of Dentistry, Autonomous University of the State of Mexico, Toluca de Lerdo 50130, Mexico; vhtoralr@uaemex.mx; 6 Cellular Biology Laboratory, Health Sciences Center, Dentistry School, Autonomous University of Baja California, Mexicali 21040, Mexico

**Keywords:** oxidative stress, impacted third molar, third molar surgery, biomarkers, reactive oxygen species, total antioxidant capacity

## Abstract

Oxidative stress (OS) has gained substantial relevance due to its involvement in the pathogenesis of numerous systemic diseases. It is characterized by an imbalance between the production of reactive oxygen species (ROS) and the capacity of endogenous antioxidant systems to neutralize them. Various factors, including trauma, immunological alterations, and psychological stress, may contribute to this condition. The aim of this narrative review was to analyze OS markers and total antioxidant capacity (TAC) in asymptomatic and pericoronitis-associated impacted mandibular third molars (ITMs). This review examines the relationship between OS and impacted ITMs, highlighting the importance of timely clinical management to prevent chronic tissue damage. Current evidence indicates that OS biomarkers such as myeloperoxidase (MPO), malondialdehyde (MDA), uric acid (UA), and nitric oxide (NO) are elevated in patients with ITMs, including those classified as asymptomatic, and that a reduction in total antioxidant capacity (TAC) has been observed. The surgical removal of ITMs can effectively reduce OS levels. Following the procedure, oxidative markers typically return to normal within a relatively short period of time, and there is often a significant improvement in TAC.

## 1. Introduction

The presence of impacted ITMs has been associated with a wide range of clinical complications. Among the most common is pericoronitis, defined as an inflammatory and infectious response affecting the soft tissues that partially or completely cover an unerupted tooth [1,2]. In addition, these molars may contribute to the development of periodontal disease in neighboring teeth and promote the onset of caries in those structures [1]. They have also been linked to the formation of odontogenic cysts and tumors. Taken together, these potential complications highlight the need for timely clinical and radiographic evaluation to determine the most appropriate management approach [2,3,4,5,6,7,8,9,10,11,12,13].

ITMs are typically clinically and radiographically identified between 14 and 25 years of age [14,15]. Epidemiological studies on pericoronitis report a prevalence ranging from 5% to 39%, and this condition is often associated with pain and trismus [16,17,18,19]. The decision to extract an ITM is influenced by the risk of causing nerve injury in the surrounding region during the surgical procedure, which is why the indication for prophylactic surgery remains a subject of ongoing debate [20,21]. In this context, the National Institutes of Health Consensus Development Conference established specific criteria for recommending the extraction of these molars [7,22]. It is important to note that after the age of 25, the likelihood of spontaneous eruption decreases significantly [23].

Current evidence suggests that asymptomatic ITMs may not be a purely dormant structure. Preliminary findings indicate that it could act as a source of subclinical inflammation, which can disrupt the homeostatic balance of the oral microenvironment [24,25]. This chronic inflammatory state promotes a redox imbalance, in which the production of ROS exceeds the neutralizing capacity of the host’s enzymatic and non-enzymatic defense mechanisms [26,27,28].

In asymptomatic patients, the retention of ITMs has been associated with a significant reduction in salivary TAC. This decrease suggests the presence of ongoing oxidative stress that potentially affects the periodontal and periapical tissues, even in the absence of clinical symptoms [29,30]. These findings indicate that rather than being inert, this latent oxidative stress might contribute to tissue degradation and could potentially increase the risk of cystic degeneration or other inflammatory complications in the follicle [31,32]. Consequently, the presence of retained ITMs is linked to a reduction in local antioxidant defenses and may modify molecular signaling pathways before any clinical signs of pain or infection appear [33,34,35].

This review aims to examine the role of oxidative stress and TAC levels, highlighting their potential as biomarkers for assessing the pathological risks associated with retained ITMs.

## 2. Search Strategy and Selection Criteria

To conduct this narrative review, a comprehensive electronic search was performed over a 10-year period, from 1 January 2015 to October 2025. The primary databases consulted were PubMed/MEDLINE, Cochrane, EMBASE, and SpringerLink. The search strategy employed specific keywords such as “Oxidative Stress” (OS), “Reactive Oxygen Species” (ROS), “Total Antioxidant Capacity” (TAC), and specific biomarkers including “Myeloperoxidase” (MPO), “Malondialdehyde” (MDA), and “Nitric Oxide” (NO), combined with terms related to “Impacted Third Molar” (ITMs) and “Pericoronitis” using Boolean operators (AND, OR).

The selection of studies focused on articles published in English that investigated the relationship between oxidative stress biomarkers and ITMs in dental follicle, saliva, or gingival tissue samples. Priority was given to studies that clearly categorized ITMs as either symptomatic (associated with pericoronitis/pain) or asymptomatic, allowing for a synthesis of data regarding inflammation severity and local redox balance. Studies involving patients with systemic inflammatory diseases, smokers, or lacking a clear clinical classification were excluded to minimize confounding factors. It is important to note that, given the narrative nature of this review and the methodological heterogeneity of the included studies, this work provides a qualitative synthesis of the evidence and does not strictly adhere to the PRISMA guidelines for systematic reviews or meta-analyses.

## 3. Basic Mechanisms of Reactive Species in Oxidative Stress

### 3.1. Definition and Redox Balance: From Physiology to Pathology

ROS and Reactive Nitrogen Species (RNS) are unstable molecules derived from oxygen and nitrogen metabolism, which, under normal physiological conditions, appear to play important roles in cellular signaling and in defending against microorganisms [26,32,33]. To maintain homeostasis, cells possess antioxidant defense systems, both enzymatic (superoxide dismutase, catalase, glutathione peroxidase) and non-enzymatic (vitamins C and E, polyphenols, among others) [28,31].

The concept of OS emerges when this balance is potentially disrupted. Various factors including trauma, emotional stress, immune alterations, or environmental pollution, may contribute to an imbalance in which the production of free radicals exceeds the capacity of antioxidant defenses [28]. This condition is often classified in dual terms: as a necessary physiological process or “oxidative eustress” (e.g., pathogen elimination), and as a pathological mechanism or “oxidative distress,” which has the potential to lead to extensive molecular damage [30].

### 3.2. Mechanisms of Molecular Damage

When antioxidant defenses are exceeded, free radicals (such as the hydroxyl radical and superoxide) may interact with critical biological structures. This interaction is not limited to an isolated reaction; rather, it can trigger cytotoxic molecular cascades: i. Lipid Peroxidation (LPO): ROS attack fatty acids in cell membranes, generating unstable products. Measuring byproducts such as MDA is considered a valuable approach for evaluating this specific form of damage, making it a key marker associated with tissue injury [31]. ii. Protein and Nucleic Acid Modification: ROS and RNS may mediate modifications that could compromise cellular function and potentially induce apoptosis or mutations [26,29].

### 3.3. OS in the Microenvironment of ITMs

The potential clinical significance of these basic mechanisms may be reflected in their function within the dental follicle and pericoronal tissues of ITMs. Chronic or acute inflammation associated with tooth retention and appears to increase innate immune activity, potentially enhancing ROS production by neutrophils and macrophages [24]. Observations suggest a correlation between the inflammatory microenvironment of ITMs and specific OS markers, such as: NO: Elevated NO levels in dental follicles of symptomatic ITMs compared with asymptomatic cases suggest that NO may act as both an inflammatory mediator and an indicator of active OS in follicular tissue [32,33]. Moreover, excessive LPO production in the tissues surrounding ITMs could contribute to molecular damage, potentially sustaining inflammation and slowing tissue healing [31,32,33].

### 3.4. Biomarkers and Clinical Relevance

To evaluate the potential impact of OS on ITM pathology, biological fluids and tissues are analyzed through TAC and specific biomarkers. In oral surgery, the saliva, gingival tissue, and the pericoronal follicle represent the primary sources for identifying these biochemical alterations [30].

**Figure 1:**
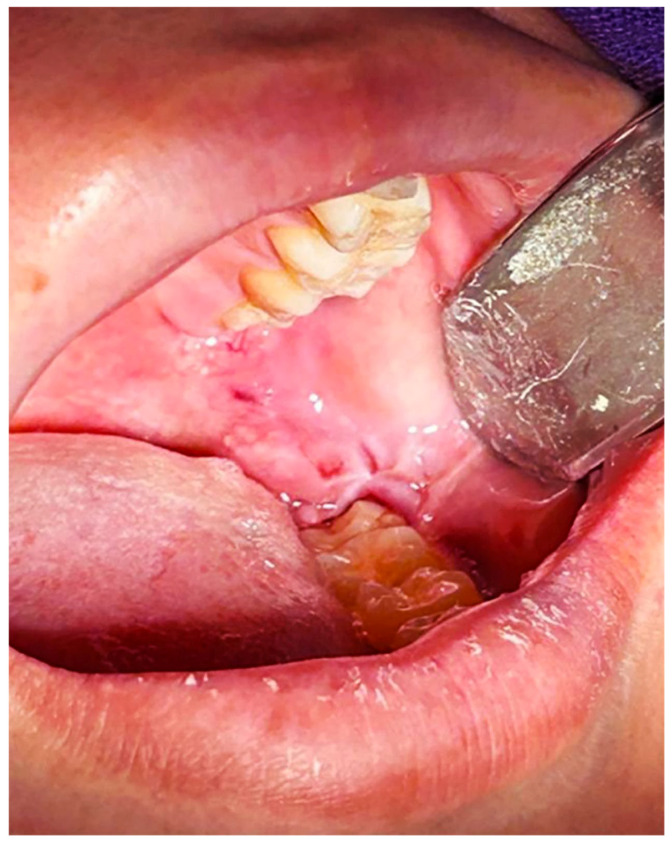
Representative clinical case of a thirty-year-old male with a clinical diagnosis of pericoronitis and type II diabetes mellitus. This image is included for illustrative purposes to depict the inflammatory condition associated with altered oxidative stress markers, specifically MPO and MDA.

Although OS has been linked to various systemic (diabetes, cardiovascular diseases) and oral conditions (periodontal disease, lichen planus) [26,27,36,37,38,39,40,41,42,43,44]. In the case of ITMs, redox imbalance could potentially manifest clinically as an enhanced inflammatory response and postoperative pain, which might be more pronounced in patients with metabolic disorders than in those without. Table 1 presents the expression of biomarkers associated with OS in oral and systemic diseases, while Figure 1 illustrates a clinical case of a patient with Type II Diabetes Mellitus and pericoronitis associated with an ITM; in this specific instance, the clinical presentation is accompanied by altered oxidative stress markers and reduced TAC.

## 4. Impacted Third Molars and Their Oxidative Stress Markers

Patients undergoing oral surgery for the extraction of ITMs exhibit transiently elevated levels of systemic inflammation, homeostatic disruptions, and increased OS parameters, which typically return to baseline levels during the postoperative healing phase [49]. Among the most widely studied OS biomarkers are MPO, MDA, NO, and UA [32,34,37].

UA, which accounts for approximately 70% of the changes in TAC, is used to estimate the health status of the tissues surrounding the third molar. It is one of the most relevant non-enzymatic antioxidants present in ITMs and is directly associated with TAC. Therefore, evaluating these biomarkers provides valuable insight into the organism’s response to oxidative damage induced by the presence of these teeth [2,3,28,32,34,37].

NO, in turn, is synthesized from L-arginine through the action of the NO synthase isoenzyme. It participates in the regulation of vascular tone, osteogenesis, and bone resorption and appears to play a key role in cellular signaling [33,34].

Other biomarkers involved in wound healing after undergoing ITMs surgery that reflect cellular damage and inflammation, likely resulting from the destructive process of alveolar bone, are lactate dehydrogenase (LDH, EC 1.1.1.27), aspartate aminotransferase (AST, EC 2.6.1.1), alanine aminotransferase (ALT, EC 2.6.1.2), acid phosphatase (ACP, EC 3.1.3.2), tartrate-resistant acid phosphatase (TRAP; EC 3.1.3.2), and alkaline phosphatase (ALP, EC 3.1.3.1) [28]. Dental follicles of clinically and radiographically asymptomatic ITMs showed increased levels of MDA compared with healthy gingival tissue of the same patient, suggesting that OS may be elevated even in asymptomatic ITMs [32,33,37]. Interestingly, high levels of MDA and MPO have also been associated with oral cancer, highlighting the potential for these molecules to mediate significant cellular damage [34,47]. The molecules involved in OS analyzed in the ITMs detected by several studies are outlined in Table 2.

## 5. ITM Surgery: Changes in the Levels of OS

The surgical removal of ITMs is the most common procedure following the onset of pericoronitis. In general, the intervention is performed after initial antibiotic and anti-inflammatory management. The surgical procedure involves the design of a mucoperiosteal flap, followed by a combination of manual and low-speed rotary instrumentation to carry out the ostectomy and odontosection [53]. Despite the extent of the ostectomy required, studies indicate that OS markers such as MDA and MPO tend to decrease after various postoperative periods [37,46].

The postoperative period spans several days and is typically characterized by pain, edema, discomfort, trismus, and dysphagia, and it may also involve complications such as infection, dysesthesia, and hemorrhage [37]. As recovery progresses, patients’ TAC has been observed to improve, in some cases reaching levels higher than those recorded preoperatively. This trend suggests a potential restoration of the antioxidant defense system [24,28]. Likewise, a significant reduction in OS parameters has been documented weeks after ITM surgery; this change may be associated with the normalization of CRP levels during the postoperative recovery [33,37,49].

## 6. Discussion

OS has long been recognized as a key factor in the pathophysiology of orofacial pain [54]. Investigating specific OS biomarkers and their signaling pathways within the context of ITMs offers a promising avenue to better understand the biological sequelae resulting from the oxidant-antioxidant imbalance. This is particularly relevant for elucidating mechanisms underlying pain perception and potential links to broader aging-related conditions [55].

Key biomarkers identified in ITM research include MDA, MPO, and NO. Comparative analyses across study groups indicate that the surgical removal of ITMs is associated with the normalization of OS levels, a recovery closely paralleled by a rebound in TAC. It is well established that elevated MDA and MPO concentrations signal cellular injury and are hallmarks of various oral pathologies [32,33,37]. Notably, high MDA levels have been correlated with malignant transformation and cystic lesions, suggesting a theoretical biological overlap with the inflammatory processes observed in prolonged ITM retention [13,47].

Nevertheless, a careful evaluation of these findings is warranted due to the methodological variability among studies. While Camacho-Alonso et al. [37] successfully validated saliva as a reliable, non-invasive proxy for follicular tissue biomarkers, specifically MDA and MPO, other studies have been limited to isolated histopathological assessments or systemic blood markers [31]. Furthermore, the interpretation of TAC and OS findings requires a cautious approach, as they may be influenced by potential confounding factors. Variables such as age, oral hygiene, dietary habits, psychosocial stress, and periodontal status play a significant role in systemic and local redox balance and should be considered when assessing the specific impact of ITMs [15,24,52,56].

An important but often underappreciated distinction lies in clinical classification: although NO levels are significantly higher in patients with a history of pericoronitis, current evidence shows that even asymptomatic ITMs exhibit baseline OS levels that exceed those of healthy controls. This biological consistency across different matrices-saliva, follicle, and blood-provides strong evidence suggesting that retained ITMs may not be biologically inert.

To mitigate OS magnitude and enhance postoperative quality of life, diverse clinical strategies have been proposed. Surgical outcomes are heavily modulated by the chosen therapeutic approach, with emerging evidence supporting the efficacy of advanced modalities like photobiomodulation and piezosurgery, alongside adjuvant therapies [50]. Furthermore, integrating antioxidant protocols into ITM management may offer protective benefits against OS-mediated cellular injury [50,52,56]. These biological findings have significant implications for clinical decision-making, adding a biochemical perspective to the traditional watchful waiting approach often applied to asymptomatic ITMs.

The persistence of OS and the continued reduction of TAC, even in the absence of overt symptoms, indicate that tooth retention imposes a chronic, subclinical burden. Consequently, the potential benefit of prophylactic extraction could be considered not only as a preventive measure against mechanical or infectious complications, but also as a therapeutic intervention aimed at potentially eliminating a silent source of oxidative activity. However, this biological rationale should be balanced against surgical risks to avoid definitive clinical generalizations.

While various antioxidant supplements, ranging from vitamin E and polyphenols to omega fatty acids, have been explored for OS control, the current body of evidence remains inconclusive regarding their efficacy [31,48,51,57]. Future research must therefore prioritize a deeper dissection of the inflammatory mechanisms linked to OS to validate targeted therapeutic strategies capable of modulating this stress response [52].

## 7. Conclusions

The main OS-related molecules involved in ITMs are MDA, MPO, and NO; following ITM surgery, their levels tend to return to normal, accompanied by an increase in TAC. From a biochemical perspective, this evidence provides a biological rationale that favors the extraction of ITMs, potentially including those with or without a history of pericoronitis [33].

The main finding of this literature review is that asymptomatic ITMs appear to contribute to a state of chronic OS. Surgical removal is associated with a reduction in the oxidative burden of biomarkers such as MPO and MDA in saliva, an effect that has been observed weeks after extraction.

The strengths of this review include the fact that we performed a comprehensive search over a ten-year period and that we highlighted saliva as a valid, noninvasive diagnostic fluid that correlates with follicular tissue markers. However, limitations exist due to the heterogeneity of the included studies and the scarcity of current evidence regarding the specific protocols used for antioxidant therapies in ITM surgery.

The mechanisms underlying OS remain poorly understood, particularly the fluctuations in biomarker levels during different stages of disease and the variations in TAC. Therefore, future research should focus on standardizing OS biomarker essays for diagnostic purposes. Establishing unified protocols based on noninvasive biological fluids, such as saliva, is essential to deepen our understanding of the role played by OS and its relationship with ITMs.

Understanding the OS processes associated with ITMs and their pathogenic mechanisms may contribute to improved clinical decision-making regarding the management of asymptomatic ITMs in the future.

The use of specialized devices, as well as pharmacological and antioxidant therapies, may represent complementary options for managing postoperative pain and inflammation, ultimately enabling ITMs surgical procedures that have a reduced impact on quality of life.

## 8. Formal Sections

This article is based on previously published studies and does not involve human or animal research.

## Figures and Tables

**Table 1:** **Biomarkers’ expression is associated with oral and systemic disease.**

Category	Disease	Biomarkers/Oxidative Stress	Mechanism of Association	Reference
**Oral** **Conditions**	Pericoronitis	NO, MDA, MPO	Acute inflammation is associated with elevated NO in the dental follicle. NO may regulate bone resorption and vascular tone, where excessive levels could indicate potential tissue damage.	[16,17,18,19,32,33]
	Periodontal Disease	ROS	The antibacterial response of neutrophils and macrophages generates a flux of ROS; it appears to share a similar mechanism of subclinical inflammation with ITMs.	[24,38,45,46]
	Caries	TAC (antioxidants)	Salivary OS has been related to the onset of caries and possible disturbances in dentin mineralization.	[1,3,4,40]
	Pulpitis/Apical Periodontitis	ROS	OS potentially contributes to the local and systemic events of pulpal and periapical inflammation.	[41,42,43]
**Premalignant** **Lesions**	Leukoplakia	ROS	Identified as a condition where a potential relationship between OS and the development of malignancy has been observed.	[24,25,26,40,41,42,43,44]
	Lichen Planus	MDA	Salivary MDA is used to evaluate OS in this chronic inflammatory condition.	[39]
**Malignant** **Lesions**	Oral Cancer	MDA, MPO	High levels of MDA and MPO are associated with cellular damage in malignant tumors. DNA oxidation is considered a factor in carcinogenesis.	[47]
**Systemic** **Diseases**	Atherosclerosis	ROS, CRP	OS may contribute to the pathogenesis of atherosclerosis. ITM surgery has been linked to the normalization of CRP levels in some studies.	[31,47]
	Diabetes Mellitus Type II	ROS	Periodontal disease and ITMs may share pathways of systemic inflammation and reactive species production with diabetes.	[26,27,48]
	Neurodegenerative	Free radicals	Free radicals have been implicated in pathogenesis; oxidative damage and metabolic dysfunction may present shared mechanisms.	[27,31]
	Hypertension	ROS	Alteration in OS balance has been observed in systemic diseases such as hypertension, suggesting a potential link to systemic inflammatory status.	[26,27]

**Abbreviations**: TAC, total antioxidant capacity; MPO, myeloperoxidase; LDH, lactate dehydrogenase; TRAP, tartrate-resistant acid phosphatase; SOD, superoxide dismutase; LPO, lipidic peroxidase; MDA, malonaldehyde; NO, nitric oxide; GSH, glutathione; UA, uric acid; E, enzymatic; NE, non-enzymatic; OS, oxidative stress; CRP, C-reactive protein.

**Table 2:** **OS molecules and biomarkers associated with ITMs.**

Molecule	Type	Diagnostic Importance	Association with ITMs	Reference
**TAC**	**E**	Indicator of the potential to withstand oxidative damage; decomposition of peroxides.	Decreases significantly immediately after surgery and progressively increases at 7 and 30 postoperative days, suggesting tissue recovery.	[37]
**MPO**	**E**	Enzyme for inflammatory regulation converts chloride ions into hypochlorous acid.	Significant increases have been observed in saliva and follicles of patients with impacted ITMs.	[37,50]
**LDH**	**E**	Indicator of cellular damage and inflammation associated with alveolar bone destruction.	Increases in saliva, likely due to bone and cellular trauma during ITM surgery.	[28]
**TRAP**	**E**	Considered a marker of osteoclast activity and bone resorption.	Detected in saliva during alveolar healing and bone remodeling phases.	[28]
**SOD**	**E**	Catalyzes the dismutation of the superoxide radical; helps protect against cellular damage.	Appears to modulate oxidative response in inflammation and ITMs healing.	[50]
**LPO**	**E**	Enzyme that plays a role in various biological processes.	Identified as a potential inflammation marker in blood and retromolar tissue in the postoperative period.	[51]
**MDA**	**NE**	Marker of lipid peroxidation that reflects potential damage to cell membrane integrity.	Elevated levels found in follicles of asymptomatic ITMs and saliva, suggesting the presence of subclinical inflammation.	[37,50]
**NO**	**NE**	Cell signaling; in excess, it may cause tissue damage, apoptosis, and bone resorption.	Significantly higher levels observed in follicles with a history of pericoronitis compared with asymptomatic ones.	[32,33]
**GSH**	**NE**	Essential molecule for cellular homeostasis; plays a fundamental role against oxidative damage.	Its preoperative preservation via antioxidants may improve homeostatic balance in ITM surgery.	[50,51]
**UA**	**NE**	Main non-enzymatic antioxidant in saliva product of purine metabolism.	Salivary levels have been observed to change after surgery, potentially influencing TAC.	[28,50,52]

## Data Availability

No new data were created or analyzed in this study. Data sharing is not applicable to this article.
